# Expert consensus on the nursing management of critically ill elderly patients with coronavirus disease 2019

**DOI:** 10.1002/agm2.12107

**Published:** 2020-04-12

**Authors:** Xia Wang, Chao Sun, Hui‐xiu Hu, Zi‐xin Wang, Hui Wang, Hua Peng, Jian‐hong Qiao, Lan Gao, Gerontological Nursing Alliance, National Center of Gerontology, Danping Cai, Meng Cai, Chuanyao Deng, Ying Deng, Yuanyuan Han, Yan Jiang, Hong Li, Youwan Liao, Ling Wang, Xinjuan Wu, Liqing Yue, Peiyu Zhao, Ting Zhou

**Affiliations:** ^1^ Department of Nursing Beijing Hospital National Center of Gerontology Institute of Geriatric Medicine Chinese Academy of Medical Sciences Beijing China; ^2^ Department of Respiratory & Critical Care Medicine Beijing Hospital National Center of Gerontology Institute of Geriatric Medicine Chinese Academy of Medical Sciences Beijing China; ^3^ Department of Nursing Tongji Hospital Affiliated to the Tongji Medical College, Huazhong University of Science and Technology Wuhan China; ^4^ Department of Nursing National Clinical Research Center for Geriatric Disorders Xiangya Hospital Central South University Changsha China; ^5^ Department of Nursing The First Affiliated Hospital of Shandong First Medical University Shandong Provincial Qianfoshan Hospital Jinan China; ^6^ Department of Neurology The First Hospital of Jilin University Changchun China

**Keywords:** critical illness, novel coronavirus, nursing, pneumonia

## Abstract

The novel coronavirus (2019‐nCoV) was first detected in patients with pneumonia of an unknown cause in Wuhan, China in December 2019. It has since been confirmed as the pathogen for the new coronavirus pneumonia, recently named “coronavirus disease 2019” (COVID‐19) by the World Health Organization. Although the general population is commonly susceptible to the disease, infected elderly people show fast progression and severe manifestations with a high proportion in critical condition as a result of compromised immunity and underlying diseases. In order to improve the quality of nursing, reduce complications, and decrease mortality of critically ill elderly patients, we assembled a national expert group with expertise in critical nursing to write this consensus, based on a literature review and a subsequent panel discussion. The consensus covers the assessment, clinical nursing, discharge care, and other aspects of care for critically ill elderly patients with COVID‐19, aiming to share insights and provide guidance for clinical practice.

## 1 INTRODUCTION

The emergence and spread of coronavirus disease 2019 (COVID‐19) has resulted in tens of thousands of pneumonia cases both inside and outside Hubei Province, China. Although the general population is commonly susceptible to the disease, infected elderly people show fast progression and severe manifestations with a high proportion in a critical condition as a result of compromised immunity and underlying diseases. The “Notice on the Prevention of the Coronavirus Disease 2019 Outbreak Among the Elderly” issued by the National Health Commission of the People’s Republic of China clearly states that “due to the high proportion of critically ill patients among the elderly, infection prevention and control should be regarded as an important mission at present to do our best to reduce critical illness and death cases.”^1^ Therefore, in order to improve the quality of nursing, reduce complications, decrease mortality of critically ill elderly patients, and provide guidance for clinical practice, we assembled a national expert group with expertise in critical nursing to write this consensus, based on a literature review, first‐line clinical experiences, and a subsequent panel discussion.

## 2 EVALUATION

### 2.1 Evaluation principles

Nurses should properly arrange critically ill elderly patients with COVID‐19 and complete the evaluation in the shortest time possible. In the meanwhile, urgent care, such as wheelchair/flatbed transfer, oxygen inhalation, electrocardiogram monitoring, sputum suction, and establishment of venous access, should be completed.

### 2.2 Evaluation content

The evaluation should include three components: general condition assessment, self‐care ability assessment, and specialized assessment. The evaluation’s specific content should be reasonably selected according to the nursing human resources available and the critical degree of the patient’s condition. 

#### 2.2.1 General condition assessment

The general condition assessment should include age, present history illness, past medical history, allergic history, vital signs, oxygen saturation, breath, consciousness state, and systemic symptoms, such as fever, cough, expectoration, chest congestion, hypodynamia, muscle soreness, and diarrhea. 

#### 2.2.2 Self‐care ability assessment

The self‐care ability assessment should include the Barthel Index for Activities of Daily Living^2^ according to which patients can be categorized into four grades: total independence (score 100), mild dependence (score between 61 and 90), moderate dependence (score between 41 and 60), and severe dependence (score <40). Nurses should determine the nursing grades according to the patient’s degree of dependence to provide appropriate nursing care. 

#### 2.2.3 Specialized assessment

The specialized assessment should include cognitive functional assessment, nutritional status assessment, and venous thromboembolism (VTE) risk assessment. The Mini‐Cog Test^3^ is recommended for the evaluation of cognitive function as it is brief, minimally influenced by language and education level, and easily accepted by patients. The Nutrition Risk in Critically Ill (NUTRIC) Scale may be used to evaluate the nutritional status of critically ill elderly patients.^4^ However, considering elderly patients are prone to suffering from malnutrition, nutritional support should be provided immediately after admission in the case of insufficient medical staff. VTE risk assessment varies depending on different clinical situations: For medical patients, the Padua Scale is recommended, while for surgical patients, the Caprini Risk Assessment Model is recommended.^5^

## 3 CLINICAL NURSING

### 3.1 Disease observation and symptom nursing

- Dynamically monitor patients’ vital signs, focusing on temperature, respiratory rhythm, rate, depth, and SpO_2_. For patients with fever, antipyretic treatment should be performed according to the doctor’s advice. After the use of antipyretic drugs, changes in temperature and sweating should be closely monitored. Moreover, for patients who sweat a lot, nurses should dry the sweat as soon as possible, change their clothes and bed units, and encourage them to drink more water. If necessary, report to the doctor for timely fluid rehydration. In addition, according to the respiratory patterns, oxygen saturation, and arterial blood gas analysis results, determine patients’ blood oxygenation statuses, and promptly correct hypoxemia.
- Dynamically monitor water electrolytes, acid‐base balance, and infection indicators of patients to determine the occurrence of complications, such as acute respiratory distress syndrome (ARDS), septic shock, stress ulcers, and deep vein thrombosis. Once any complications occur, nursing routines and measures of corresponding complications should be implemented.
- Observe patients’ consciousness states and systemic symptoms, such as cough, expectoration, chest tightness, dyspnea, and cyanosis. Once the patient has the above symptoms, implement oxygen therapy following the doctor’s advice and observe the effect. The patient’s condition should be reported to the doctor in time to adjust the oxygen flow rate or oxygen therapy method. Also, the 24‐hour intake and output volume should be recorded in the nursing document.
- For elderly patients, special attention should be paid to strengthen the observation and nursing of their underlying diseases and monitor relevant indicators, such as hypertension, diabetes, cardiovascular disease, and cerebrovascular disease.

### 3.2 Oxygen therapy

For critically ill elderly patients with COVID‐19, respiratory support techniques should be selected individually according to the degree of hypoxia, tolerance, and the doctor’s advice. Nasal catheter, mask, high‐flow nasal cannula (HFNC), and noninvasive ventilation may be the preferred respiratory support techniques for patients with mild to moderate hypoxia, while noninvasive ventilation, invasive ventilation, and extracorporeal membrane oxygenation (ECMO) are recommended for patients with moderate to severe hypoxia.^6^ As older patients often have underlying lung diseases, they are prone to suffering from carbon dioxide retention. Therefore, when necessary, the patient’s blood gas should be reviewed and the oxygen therapy should be changed. Recommendations for oxygen therapy and nursing care for critically ill elderly patients with COVID‐19 are as follows.

#### 3.2.1 Nasal catheter and mask oxygen inhalation

When nasal catheter oxygen inhalation is used, the oxygen flow rate is generally <5 L/min. Disposable nasal oxygen catheters and humidification devices are recommended. The front end of the nasal oxygen catheter should be placed in the patient’s nostril with a depth of 1.5 cm, and the patient should wear a surgical mask to prevent aerosol diffusion.^4^ A water‐based lubricant can be used if the nasal mucosa is dry, but not petroleum jelly or other oil‐based agents. When mask oxygen inhalation is used, the oxygen flow rate is usually set at 5‐10 L/min and disposable nasal oxygen catheters and humidification devices are recommended. The mask should completely cover the patient’s nose and mouth to reduce aerosol diffusion, but a surgical mask is not necessary.^6^ During oxygen inhalation by nasal catheter or mask, the patient’s breath and oxygen saturation should be closely observed and arterial blood gas analysis should be implemented if necessary. If oxygen therapy persistently fails to reach the target or carbon dioxide retention occurs, nurses should inform the doctor immediately and change oxygen therapy methods if necessary.

#### 3.2.2 HFNC

In order to reduce the diffusion of aerosol and droplets, when starting HFNC, the operation should be conducted in the following sequence: (1) start up, (2) set initial parameters, (3) fit the nasal plug to the patient, and (4) deliver oxygen. When stopping, the sequence of operation is to shut down or reduce the oxygen flow to zero and then remove the nasal plug. Considering that critically ill elderly patients with COVID‐19 may get worse at any time, when HFNC is used or stopped, the oxygen supply should not be interrupted and some necessary devices needed for nasal catheter oxygen inhalation, noninvasive ventilator, or trachea intubation should be prepared in advance according to the condition. Keep the device and pipeline at or below the level of the patient’s head to avoid choking or asphyxiation caused by backflow of the condensation in the pipeline. In the process of HFNC, pay close attention to the application effect. If there is no improvement in oxygenation indicators or if they deteriorate after 2 hours of use, the doctor should be informed immediately and a change to noninvasive mechanical ventilation should be considered.^4^

#### 3.2.3 Noninvasive mechanical ventilation

Before initiating noninvasive mechanical ventilation, patients should be fully informed to eliminate resistance and so they can be taught to inhale through the nose. In order to reduce aerosol diffusion, an oral‐nasal mask is recommended but not a nose mask. If conditions permit, a disposable breathing valve is recommended instead of a mask‐integrated valve and platform valve. Filters can be added between the mask and the breathing valve and having the vantage facing the operator should be avoided.^7^ In the process of using, adjust the parameters of inspired positive airway pressure, expired positive airway pressure and oxygen concentration according to the doctor’s advice, and closely observe the patient’s consciousness, oxygenation, patient‐ventilator synchrony, and improvement of respiratory function. If the condition does not improve in a short time, or signs of deterioration (such as consciousness disorders or coma) occur, the doctor should be informed immediately and a change to invasive mechanical ventilation should be considered.

#### 3.2.4 Invasive mechanical ventilation

It is recommended that patients with nasal trachea cannula or tracheotomy should wear surgical masks to avoid aerosol diffusion. Disposable respiratory loops are recommended, and regular replacement is not recommended unless contamination is apparent.^7^ Pay close attention to ventilator operation, oxygenation index, pipe fixation of trachea cannula or tracheotomy, and sputum drainage during use. If endotracheal suction is required, closed endotracheal suction systems should be selected (see “Airway management” section below for details). As elderly patients are prone to comorbid ventilator‐associated pneumonia, when using noninvasive mechanical ventilation, the head of the bed must be raised by 30°‐45°. The condensate must be removed regularly and disposed of in accordance with infectious body fluids. During the process of disposal, the accidental spilling or backflow of the condensate into the airway of the patient should be avoided.^6^, ^7^

#### 3.2.5 ECMO

During the operation of ECMO, the patient’s condition, hemodynamic changes, vital signs, oxygen saturation, arterial blood gas, central venous pressure, and other indicators should be closely observed and, if possible, invasive blood pressure should be continuously monitored. Radiator temperature, speed of pump, and auxiliary flow can be adjusted according to the indicators. Monitor the coagulation function according to the doctor’s advice every day; adjust the dosage of heparin; pay close attention to the operation of the machine; and observe whether there is abnormal vibration, translocation, looseness, curve, traction, and whether there is air intake and thrombus in the pipeline.

### 3.3 Airway management

#### 3.3.1 Artificial sputum excretion

Postural drainage, regular turning over, and patting of the back can be used to help patients with secretion retention or difficulty in sputum excretion. A vibrating sputum extractor should be avoided as much as possible, so as not to cause the risk of blood oxygen saturation decline and arrhythmia. During sputum excretion, nurses should pay attention to the patient’s complaint of discomfort, and immediately monitor the changes of vital signs and blood oxygen saturation.

#### 3.3.2 Airway atomization

Atomization treatment is a high‐risk procedure for suspected or confirmed patients with COVID‐19. Therefore, this procedure should be performed in a well‐ventilated room with a minimum number of people that can meet the patient’s care needs.^8^ Avoid using a small‐volume nebulizer, which easily causes aerosol diffusion, as much as possible. Atomization devices, such as atomized drug storage devices, breathing pipes, and atomized masks, should be disposable.

#### 3.3.3 Airway humidification

During invasive, noninvasive, or artificial airway ventilation, airway humidification can reduce the inflammatory response caused by mechanical ventilation. For reasonable airway humidification, one should avoid excessive or insufficient humidification. It is necessary to implement sputum suction immediately according to the characteristic changes of airway secretions to prevent the occurrence of emergencies, such as sputum obstruction and suffocation.

#### 3.3.4 Endotracheal suction

For patients with artificial airways, closed endotracheal suction systems should be used strictly following the principle of sputum suction on demand to avoid constant suction leading to severe cough, airway wall mucosal edema, or even bleeding.^6^ Shallow sputum suction is recommended and patients should be given 2 minutes of pure oxygen before that. In addition, each sputum suction duration should be <15 seconds and continuous negative pressure suction should be applied with pressure maintained between 80 mmHg and 150 mmHg (1 mmHg = 0.133 kPa). The closed tracheal suction catheter should be changed every 72 hours to reduce further infection caused by colonization of the tip bacteria.

### 3.4 Medication management

For critically ill elderly patients with COVID‐19, the total infusion volume should be controlled according to the patient’s condition; the infusion sequence should be reasonably arranged and the infusion apparatus should be replaced as needed. After the use of medication, observe the efficacy closely and pay special attention to the occurrence of drug‐related adverse reactions.

#### 3.4.1 Antiviral drugs

Antiviral drugs should be given to patients according to the doctor’s advice. Considering that lopinavir/ritonavir may cause diarrhea, nausea, vomiting, liver injury, myocardial injury, and other adverse reactions, special attention should be paid to monitoring the liver function, cardiac function of patients during the clinical application, and gastrointestinal reactions that may occur, requiring symptomatic treatment and care.

#### 3.4.2 Glucocorticoids

Glucocorticoids treatment should be performed in strict accordance with the prescribed dose, frequency, and duration of medication, and the dosage should be reduced gradually according to symptoms and body temperature. Attention should be paid to observe the occurrence of any drug‐related adverse reactions, such as secondary infection, ulcers, hypertension, blood glucose increase, and blood potassium decrease.

#### 3.4.3 Antibiotics

During the application of antibiotics, it is necessary to observe the occurrence of intestinal flora disorders, drug allergic reactions, fungal infections, and so forth, and to deal with possible adverse drug‐related reactions according to the doctor’s advice.

#### 3.4.4 Other drug treatments

Patients can be treated with human immunoglobulin according to the doctor’s advice. For patients with rapid progression, severe or critical disease, convalescent plasma therapy may be used.^9^ During the use, it is necessary to pay attention to the occurrence of transfusion reaction and observe the curative effect.

### 3.5 Complications nursing

As critically ill elderly patients with COVID‐19 often have coexistence of multisystem diseases, it is necessary to pay close attention to the occurrence of complications and carry out active prevention, treatment, and nursing on the basis of symptomatic treatment.

#### 3.5.1 Respiratory failure

Critically ill elderly patients with COVID‐19 are prone to dyspnea and/or hypoxemia due to the combination of multiple underlying diseases, and severe cases may rapidly progress to ARDS. When patients with moderate or severe ARDS need to be treated with invasive mechanical ventilation combined with a prone position, the nurse should follow the standard operating procedure of the prone position and change the body position by axial rotation. Meanwhile, complications, such as pressure injury, falling off the bed, pipeline slip‐off, and eye compression, should be prevented. Those patients with poor efficacy should receive ECMO treatment as soon as possible.

#### 3.5.2 Cardiovascular complications

The occurrence of acute left heart failure and myocardial injury should be monitored vigilantly in elderly patients under a variety of pathogenic factors. Especially for elderly patients with underlying heart disease, it is necessary to be cautious about fluid supplementation to avoid large amounts of rapid fluid supplementation leading to acute left heart failure. Nurses should pay close attention to changes in breathing, blood pressure, heart rate, and heart rhythm of elderly patients, and promptly identify symptoms such as dyspnea, severe wheezing, arrhythmia, and decreased blood pressure. In addition, relevant laboratory tests of b‐type brain natriuretic peptide, electrolytes, and markers of myocardial injury should be conducted, and active treatment should be coordinated with doctors. Furthermore, adjust the patient’s position, maintain unobstructed venous access, and use drugs in accordance with the doctor’s advice. Rescue supplies and medicines should be prepared and kept by high‐risk patients’ beds.

#### 3.5.3 Acute kidney injury

Treatment with anti‐infective drugs and mechanical ventilation increase the risk of acute kidney injury in elderly patients with COVID‐19. When early kidney injury manifestations (such as decreased urine volume, facial edema, and limb edema) appear, it is necessary to communicate with the doctor immediately to adjust the treatment plan. During the oliguria phase, the infusion volume and drip rate should be controlled (30 drops/min), and attention should be paid to the occurrence of hyperkalemia. During the polyuria phase, the patient’s vital signs, consciousness, appetite, and skin elasticity should be closely observed and oral rehydration salts can be used to maintain electrolyte balance. In the course of treatment, daily living nursing should be strengthened to keep the perineum clean and reduce the possibility of urinary system infection.

#### 3.5.4 Deep vein thrombosis

The risk of VTE in elderly patients with severe disease is significantly increased due to the combination of: the release of a large number of inflammatory mediators; the application of hormones and immunoglobulin resulting in high blood coagulation, coupled with mechanical ventilation; and central venous catheterization, surgery, and other operations leading to vascular endothelial injury. Therefore, all critically ill elderly patients with COVID‐19 should be treated with VTE prevention if there are no contraindications.^5^ Prevention of VTE usually includes subcutaneous injection of anticoagulant prophylaxis and mechanical prophylaxis (intermittent pneumatic booster pump prophylaxis). During the use of anticoagulant drugs, the risk of VTE and bleeding must be dynamically assessed. In particular, it is necessary to observe whether there is any bleeding or abnormal coagulation function after the use of anticoagulant drugs. Once any abnormal situation occurs, immediately inform the doctor to stop the drug and deal with it.

#### 3.5.5 Pressure injury

Because the skin of the elderly is atrophic and thin, and the senses are insensitive, it is necessary to strengthen the skin care of elderly patients to prevent pressure injury. For patients with oxygen therapy or noninvasive ventilation, the skin blood circulation of the face in contact with the mask or catheter should be regularly observed and preventive dressings, such as foam dressings, should be used to provide protection when necessary to avoid pressure injuries on the nose, face, lips, and behind the ears. Patients with diarrhea, frequent stools, or incontinence need to be alert to the occurrence of incontinent dermatitis. When antipyretic drugs are required, suppositories should be used less frequently to reduce the risk of skin damage.

#### 3.5.6 Consciousness disturbance

Some patients with COVID‐19 have symptoms similar to intracranial infections, such as headache, epilepsy, and disturbance of consciousness, and some even take intracranial infection as the first symptom. Therefore, the nurse should closely observe the patient’s neurological symptoms and, if necessary and according to the doctor’s advice, give sedative and analgesic drugs, and give reasonable physical restraints, so as to ensure the patient’s life and safety of treatment and nursing.

#### 3.5.7 Secondary infection

It is important to provide patients with oral care, nursing of various pipelines, and defecation and urination nursing. Strictly implement the aseptic operation and disinfection and isolation standards to prevent ventilator‐related pneumonia, catheter‐related bloodstream infection, catheter‐related urinary tract infection, and other secondary infections.

### 3.6 Nutritional care

It is necessary to dynamically evaluate the nutritional risk of patients, pay close attention to their nutritional status, and provide timely and reasonable nutritional support. For patients who need nutritional treatment, follow the principle of diet priority, oral route priority, and enteral nutrition (EN) priority.^10^

#### 3.6.1 Oral feeding

(1) For patients who can eat by mouth, dietary nutritional education should be done to inform patients of the importance of a reasonable diet in promoting rehabilitation and to obtain the maximum degree of cooperation from the patients. (2) A high‐protein, carbohydrate‐rich diet is recommended, especially for the elderly; easily digestible food is recommended. (3) Close attention should be paid to the nutritional status of patients, and nutritional indicators, such as albumin and ferritin, should be monitored. When oral feeding fails to meet the 60% target energy requirements of patients, EN should be started as soon as possible.^10^

#### 3.6.2 Enteral nutrition

(1) For patients with EN by nasogastric tube, the temperature of the nutrient solution should be maintained at 38°C‐42°C and continuous and uniform pumping is recommended, starting at 20‐30 mL/h, and increasing by 10 mL/h after 2 hours if no retention, until 60‐100 mL/h.^10^ It is recommended that gastric residual volume be monitored every 4 hours for gastric retention.^11^ (2) During the EN process, patients should be observed for the presence of symptoms of gastrointestinal intolerance, such as nausea, vomiting, and diarrhea. If the above intolerance symptoms appear, promptly inform the doctor and consider the use of gastrointestinal motility drugs or the post‐pyloric feeding route, such as nasointestinal tube. (3) The patient’s head should be raised 30°‐45° during feeding to reduce the risk of aspiration and aspiration pneumonia.^10^ (4) The type, speed, and quantity of EN nutrient solution should be recorded, and nutritional indicators, such as albumin, prealbumin, and ferritin, should be monitored.

#### 3.6.3 Parenteral nutrition

Supplemental parenteral nutrition (PN) is recommended as early as possible if EN fails to meet the 60% target energy and protein requirements within 48‐72 hours.^12^ The PN of critically ill patients with COVID‐19 is usually infused by central venous catheterization or central venous catheterization through peripheral venipuncture. The speed should not exceed 200 mL/h, and intravenous infusion pump is recommended.^10^

### 3.7 Psychological nursing

Critically ill elderly patients with COVID‐19 are more likely to have psychological panic and anxiety due to insufficient knowledge of the disease and lack of access to information. The following measures should be considered in these cases.

- Evaluate the patient’s cognitive changes, emotional responses, and behavior changes, and provide appropriate emotional support. For patients with anxious and depressed tendencies, self‐rating scales, such as an anxiety self‐rating scale and a depression self‐rating scale, can be used for assessment, and professional psychological personnel can be asked to help patients according to the situation.
- Various means, such as cognitive behavioral therapy, positive psychology, explaining COVID‐19 in simple and understandable language, and providing continuous information support, may help elderly patients to a timely transition to the psychological stage of treatment, and to build up the confidence to overcome the disease.
- Relaxation training, such as meditation, hypnosis, music therapy, and other ways to relieve patients’ anxiety and depression, should be provided if possible.

## 4 DISCHARGE NURSING

### 4.1 Discharge preparation

For discharged patients, conditional hospitals should set up special discharge channels with bathrooms inside. The first thing for patients after leaving the ward is to enter the bathroom of the discharge channel for personal hygiene disposal. Nurses should provide assistance for elderly patients with poor self‐care ability in bathing and changing into clean clothes. For patients’ personal belongings, discard all that can be discarded. For belongings that must be kept, 1000 mg/L chlorine‐containing disinfectant spray or 75% ethanol wiping disinfection is recommended. For clothing that must be kept, garments should be irradiated 1 day in advance with ultraviolet light for 1 hour on both sides.

### 4.2 Discharge instructions

Discharge should consist of the following: (1) Select appropriate breathing rehabilitation exercises and teach them to patients, such as airway clearance training, breathing exercises, pursed‐lip breathing, and abdominal breathing. (2) Advise patients to strengthen nutritional support, eat more high‐protein, high‐vitamin, high‐calorie food, and more fresh vegetables, fruits, milk, and so on. Likewise, instruct patients to work and rest regularly and to get adequate sleep. (3) It is recommended that patients continue to be monitored for 14 days after discharge and continue to wear masks. If possible, it is recommended to live in a single room with good ventilation, reduce close contact with others, eat separately, carry out hand hygiene thoroughly, and avoid outdoor activities. (4) It is recommended that patients return to the hospital for reexamination 2 and 4 weeks after discharge.^9^ During the period after discharge, if the patient has fever, dyspnea, or the reappearance of other uncomfortable symptoms, or if a family member with close contact has a new novel coronavirus infection or suspected infection symptoms, they should go to the hospital immediately.

## CONFLICTS OF INTEREST

Nothing to disclose.

## APPENDIX A

Authors: Wang Xia, Sun Chao, Hu Huixiu, Wang Zixin, Wang Hui, Peng Hua, Qiao Jianhong, Gao Lan, China Gerontological Nursing Alliance, National Center of Gerontology Expert Committee. Expert committee members in alphabetical order: Ting Zhou (Department of Neurology, National Center of Gerontology), Meng Cai (Department of Infection Prevention and Control, National Center of Gerontology), Chuanyao Deng (Department of ICU, the First Affiliated Hospital of Shandong First Medical University, Shandong Provincial Qianfoshan Hospital), Ying Deng (Department of Neurology, National Center of Gerontology), Yuanyuan Han (Department of ICU, National Center of Gerontology), Yan Jiang (Department of Nursing, West China Hospital, Sichuan University), Hong Li (Department of Nursing, Fujian Provincial Hospital), Youwan Liao (Department of Geriatrics, Guangdong Provincial People’s Hospital), Ling Wang (Department of Nursing, Peking University People’s Hospital), Xinjuan Wu (Department of Nursing, Peking Union Medical College Hospital), Liqing Yue (Department of Nursing, Xiangya Hospital of Central South University), Peiyu Zhao (Department of Nursing, China‐Japan Friendship Hospital), Ting Zhou (Department of Thoracic Surgery, National Center of Gerontology).

## References

[1] National Health Commission of People’s Republic of China . Notice on the prevention of the coronavirus disease 2019 outbreak among the elderly; 2020 National Health Commission website. http://www.nhc.gov.cn/xcs/zhengcwj/202001/96e82ba8a14d41b283da990d39771493.shtml. Accessed February 13, 2020.

[2] Hu J , Yang LP , Jin XQ , et al. The ADL and risk of fall of elderly hospitalized patients with different sex and nationalities. Chin J Gerontol. 2016;36(11):2745–2746.

[3] Milian M , Leiherr AM , Straten G , et al. The mini‐cog versus the mini‐mental state examination and the clock drawing test in daily clinical practice: screening value in a german memory clinic. Int Psychogeriatr. 2012;24(5):766–774. 22172089 10.1017/S1041610211002286

[4] Respiratory Critical Medicine Group of Chinese Medical Association Respiratory Medicine Chapter, Critical Care Medical Working Committee of Chinese Medical Doctor Association Respiratory Doctor Chapter . Expert consensus on the clinical application of HFNC among adults. Chin J Tuberc Respir Dis. 2019;42(2):83‐91.

[5] Chinese Medical Association Respiratory Medicine Credit Society Pulmonary Embolism and Pulmonary Vascular Disease Group, Committee on Pulmonary Embolism and Pulmonary Vascular Disease , Respiratory Physicians’ Branch of the Chinese Physicians’ Association , National Collaboration Group on Pulmonary Embolism and Pulmonary Vascular Disease Prevention and Control et al. The prevention and treatment recommendations of venous thrombosis related to 2019‐nCoV. Natl Med J China. 2020;100:E007.

[6] Respiratory Critical Medicine Group of Chinese Medical Association Respiratory Medicine Chapter, Critical Care Medical Working Committee of Chinese Medical Doctor Association Respiratory Doctor Chapter . Recommendations for airway management for adult patients with severe COVID‐19. Natl Med J China 2020;100:E004.

[7] Ge HQ , Dai B , Xu PF et al. Expert consensus on the management of infection related to the use of ventilator among patients with COVID‐19. Chin J Respir Crit Care Med. 2020;19(2):1‐4.

[8] Ni Z , Luo FM , Wang JM et al. Recommendations for atomized inhalation therapy in patients with coronavirus disease. Chin J Respir Crit Care Med. 2020;19(2):1‐6.

[9] Medical and Hospital Authority of the National Health Commission of People’s Republic of China . Notice on the issuance of the treatment programme for COVID‐19, 6th edn; 2020 National Health Commission website. http://www.nhc.gov.cn/yzygj/s7653p/202002/8334a8326dd94d329df351d7da8aefc2.Shtml. Accessed February 19, 2020.

[10] Chinese Medical Association Enteral and Parenteral Nutrition Chapter . Expert advice on enteral and parenteral nutrition therapy in patients with severe COVID‐19. Natl Med J China. 2020;100:E009.

[11] Reintam Blaser A , Starkopf J , Alhazzani W et al. Early enteral nutrition in critically ill patients: ESICM clinical practice guidelines. Intensive Care Med. 2017;43(3):380‐398. 28168570 10.1007/s00134-016-4665-0 PMC5323492

[12] Chinese Medical Association Enteral and Parenteral Nutrition Chapter . Chinese expert consensus on adult supplementary intestinal nutrition. Chin J Gastrointest Surg. 2017;20(1):9‐13.

